# First Case Report of Metastatic Squamous Cell Carcinoma of Lung Associated with Mounier-Kuhn Syndrome and Review of Literature

**DOI:** 10.7759/cureus.1394

**Published:** 2017-06-26

**Authors:** Fatima Ayub, Muhammad W Saif

**Affiliations:** 1 Internal Medicine, Rawalpindi Medical College, Rawalpindi, Pakistan; 2 Hematology/Oncology, Tufts Medical Center

**Keywords:** congenital tracheobronchomegaly, lung cancer, squamous cell cancer, mounier-kuhn syndrome, tracheal dilation, respiratory tract infection

## Abstract

Mounier-Kuhn syndrome is a relatively rare condition, mostly involving the trachea and main stem bronchi. It is caused either by the atrophy of elastic fibers or faulty fetal development of cartilage and smooth muscles, hence leading to an overall increase in the diameter of lower respiratory tract. No certain etiology was found in the majority of cases reported previously, however, several other connective tissue diseases have also been implicated with the congenital tracheobronchomegaly. One anecdotal case report mentioned the development of lung malignancy in a patient who had previously received external beam radiotherapy. Herein, we report the first case of Mounier-Kuhn syndrome in a 62-year-old male with a recent diagnosis of metastatic squamous cell carcinoma (SCC) of the lung.

## Introduction

Mounier-Kuhn syndrome, commonly known as tracheobronchomegaly, is a rare clinical and radiographic condition characterized by marked tracheobronchial dilation and recurrent lower respiratory tract infections [1]. The syndrome was first described by Mounier-Kuhn in 1932 [2]. This increase in the tract’s patency progresses further during inspiration while collapses during expiration due to the underlying loss of elastic tissue. The resulting weakness in the intercartilaginous membranes can lead to saccular outpouchings with the possible retention of respiratory secretions. This can create small zones of mucus plugs predisposing the patient to recurrent secondary suppurative infections both in the upper and lower respiratory tracts [3-4]. There are three subtypes of this syndrome [1]:

Type 1: Slight symmetric dilation in the trachea and main bronchi.

Type 2: Dilation and diverticula are distinct.

Type 3: Diverticular and saccular structures extend to the distal bronchi.

The diagnosis of Mounier-Kuhn syndrome is established with the use of computed tomography (CT) and bronchoscopy, as well as pulmonary function tests (PFTs) [5-6]. Patients may be asymptomatic; however, symptoms can range from minimal with preserved lung function to severe respiratory failure. Complications may include bronchiectasis, recurrent lower respiratory infections, recurrent pneumonias, and fibrosis [10]. Herein, we report a case of a patient with Mounier-Kuhn syndrome who developed squamous cell carcinoma (SCC) of the lung. We believe that this is the first case report of its kind.

## Case presentation

A 62-year-old man presented to the Emergency Department (ED) with the complaints of acute worsening of shortness of breath and chronic productive cough. He had been experiencing exertional dyspnea for quite some time but it progressively worsened over the past two days. It was also accompanied with mild dysphagia, hoarseness, and mild facial swelling. The patient also reported rhinorrhea and mild watery discharge from his right eye with some blurry vision. He denied fever, chills, sore throat, any change in the intensity of his cough or appearance of sputum. The patient was a known case of Mounier-Kuhn syndrome (congenital tracheobronchomegaly). Other major illnesses in his past medical history included chronic obstructive pulmonary disease (COPD), recurrent pneumonias, hypertension and hypersensitivity lung disease. He was a non-smoker and a non-alcoholic. Family history was insignificant.

The physical examination showed a cooperative patient with difficulty in breathing. His vitals were stable. His head and neck examination revealed bilateral conjunctival injection, mild watery discharge from the right eye, hoarseness and mild facial swelling. Chest auscultation showed diffuse bilateral coarse crackles and bronchial breath sounds in the upper lobe of the right lung. His heart and abdominal examination was unremarkable except for the mild right upper quadrant (RUQ) tenderness without any obvious guarding or rebound. His oxygen (O_2_) saturation was 97% on room air. Ear, nose and throat (ENT) assessment showed full and redundant nasopharyngeal and oropharyngeal soft tissue with no evidence of laryngeal edema on the flexible laryngoscopy. There was no evidence of airway compromise or stridor at that time and the patient had no difficulty of speech. His chest X-ray did not reveal any new abnormalities or consolidation when compared to his previous X-rays, thereby, ruling out COPD exacerbation or pneumonia. Laboratory data was within normal limits except for a Na^+ ^level of 130 mg/dl and hemoglobin of 11.2 g/dl. Sputum cultures were negative. However, acid fast bacilli (AFB) culture came out to be positive for mycobacterium avium complex (MAC).

**Figure 1:**
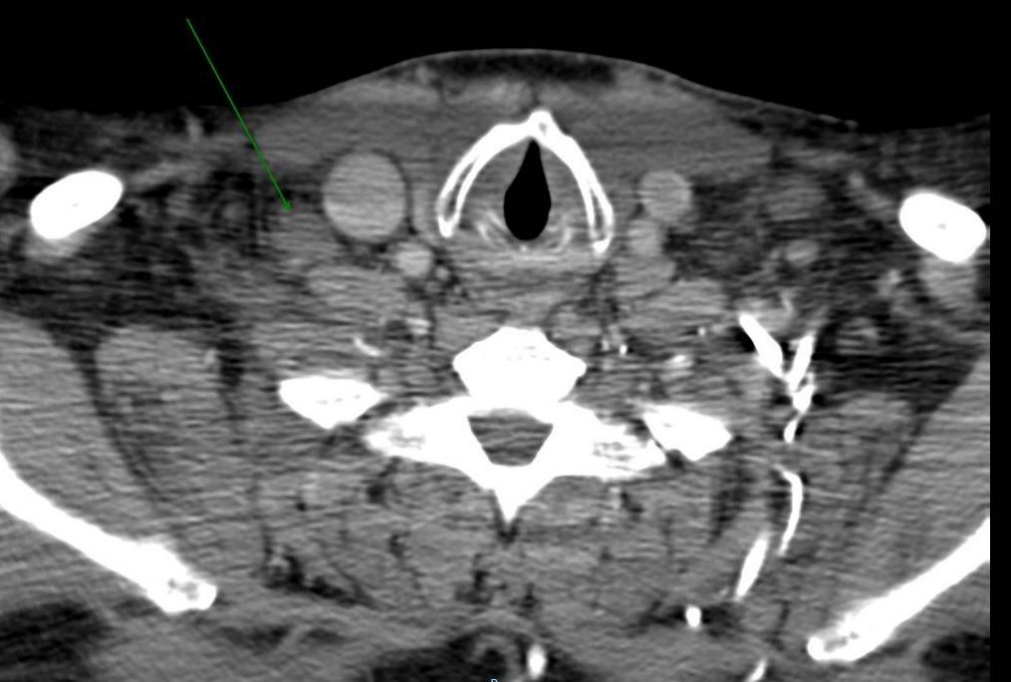
Computed tomography (CT) scan of the chest. The arrow in the scan shows a soft tissue mass that encases and compresses the superior vena cava and right posterior trachea originating from the right lung apex. The tracheal diameter is overall enlarged. Bilateral hilar lymphadenopathy is also evident, but appreciated more on the right side.

Initial evaluation with the chest CT revealed a soft tissue mass extending across the anteroposterior dimension of the right-sided mediastinum that encased and compressed the superior vena cava to approximately 2 mm in diameter (Figure 1). This led to the initial diagnosis of superior vena cava syndrome (SVC syndrome). The trachea also appeared enlarged consistent with his history of Mounier-Kuhn syndrome (Figure 1). CT scan also showed an 8.4 cm anteroposterior (AP) × 6.8 cm transverse × 5.3 cm well-marginated centrally homogenous collection in the right lung apex with nodular thickening of the right upper lung pleura (Figure 1). Further workup with right pleural biopsy resulted in securing the diagnosis of SCC of the lung. Immunohistochemical stains showed the tumor cells to be positive for the pankeratin, CK5/6, CK7 and p63 tumor markers, which was contingent with the diagnosis of SCC.

**Figure 2:**
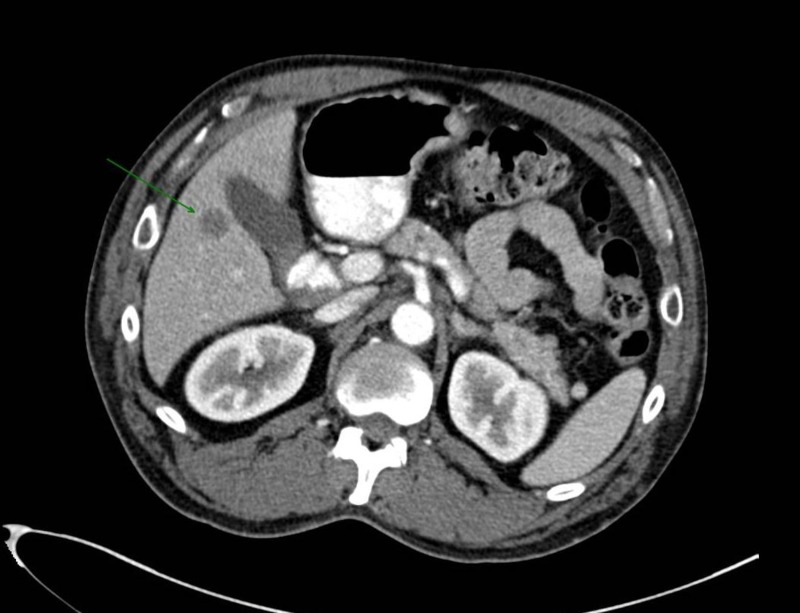
Computed tomography (CT) scan of the abdomen. The arrow indicates a 4 mm hypoattenuating lesion in the liver, which along with the other smaller ones, reflects metastatic disease in the setting of a primary squamous cell carcinoma of the lung.

**Figure 3:**
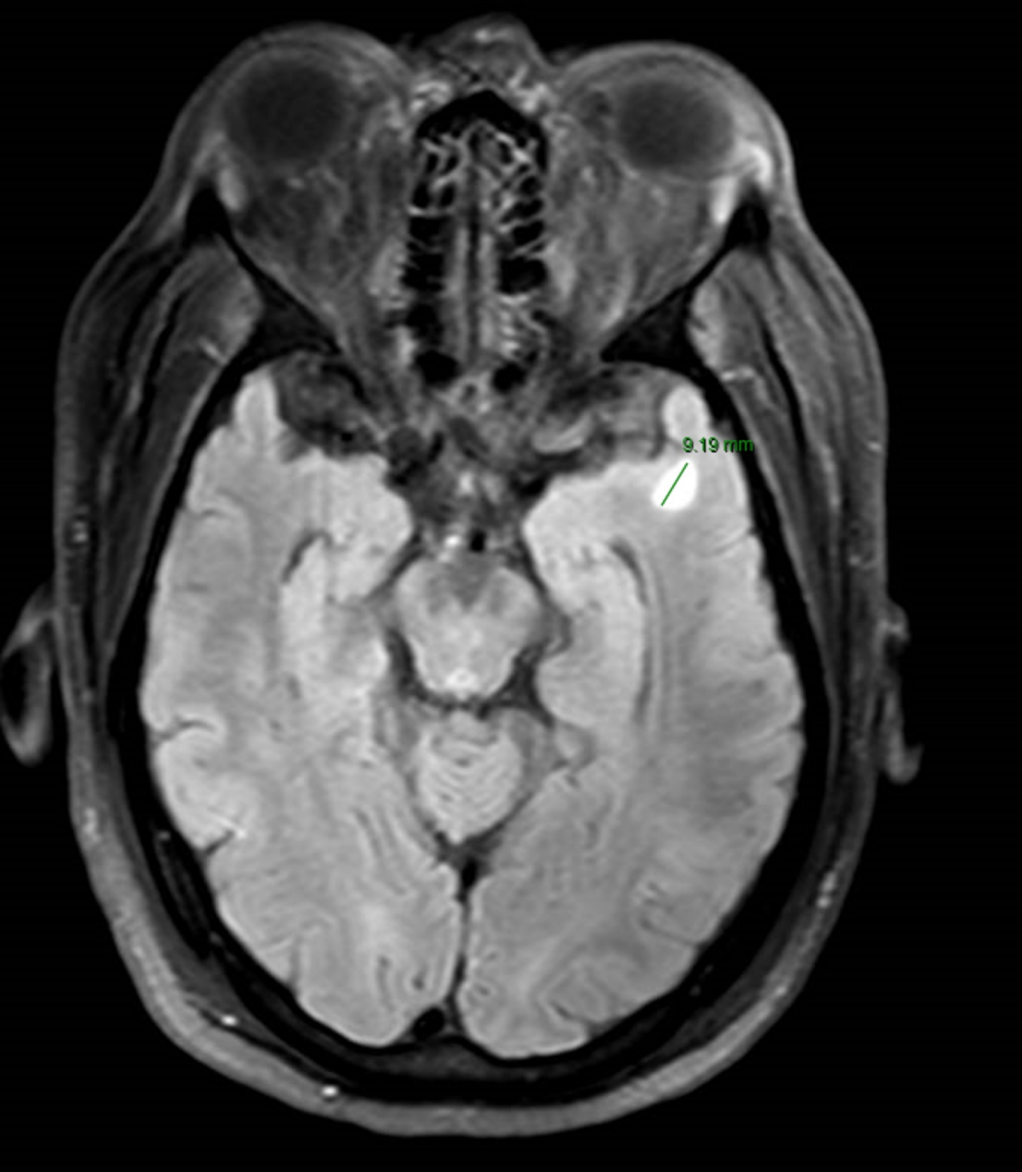
Magnetic resonance imaging (MRI) of the brain. The arrow in the scan depicts a non-enhancing lesion in the left temporal lobe of the brain, which reflects either a low-grade primary brain malignancy or a metastatic disease.

It was designated as stage IV based on the abdominal CT scan findings that showed two ill-defined hypoattenuating lesions in the liver and a slightly more well-defined 4 mm lesion in the hepatic segment 4 (Figure 2). Magnetic resonance imaging (MRI) of the abdomen confirmed these lesions to be compatible with a metastatic disease. MRI of the head showed a 9 mm non-enhancing left temporal lobe lesion and differentials included metastatic lesion, gliosis or a primary brain neoplasm due to its non-enhancing nature (Figure 3).

Treatment was started immediately after excluding pneumonia with intraveous steroids and external beam radiotherapy (XRT) over the course of 10 days (summarized in Table 1), that resulted in a substantial clinical improvement of his obstructive symptoms due to SVC syndrome. He was discharged and scheduled to receive further systemic chemotherapy as an outpatient.

**Table 1:** Treatment regimen of our patient. RUL: Right upper lobe, Gy: Gray units, RF: Right fissure.

TREATMENT SITE	PRESCRIBED		ADMINISTERED	
	Total dose (Gy)	#Fx	Total dose (Gy)	#Fx
RUL Lung	3	1	3	1
RUL Apical Lung	18	6	18	6
RF RUL Apical Lung	9	3	9	3

## Discussion

Mounier-Kuhn syndrome is an extremely rare abnormality of the lower respiratory tract, first described clinically and radiographically by Mounier-Kuhn in 1932 [1-2]. The incidence is particularly high in the African American descent. About 128 cases were reported till 2014 with an 8:1 male predominance due to unidentified factors. It mostly manifests in middle-aged men with the mean age of 53.9 years [7-8], but it is reported in the elderly too, as already depicted in our patient.

The marked airway dilation, either due to the aberrant development of connective tissue or complete absence of the smooth muscle cells of the tract results into laxity of the trachea and central bronchi [3]. Peripheral bronchi, i.e., the airways distal to the fourth-order and fifth-order divisions are usually spared [4]. The resulting intrinsic weakness in the tracheal wall can contribute to the development of diverticula and outpouchings with the retention of secretions due to the impairment of mucociliary clearance. This constellate of events can predispose to chronic pulmonary suppuration, emphysema, pneumonia, COPD exacerbation, bronchiectasis and even malignancy, either primary or metastatic [10]. Patients usually present with shortness of breath, cough and increased sputum production, occasional hemoptysis and progressive dyspnea ultimately culminating in respiratory failure. Sometimes they can also present with atypical symptoms like dysphagia, syncope, facial and upper limb edema. All these signs and symptoms can be due to a superimposed lung cancer too as diagnosed in our patient who initially presented with SVC syndrome. The overlapping symptoms of these lung diseases and the Mounier-Kuhn syndrome make the diagnosis even more challenging. It is recommended to thoroughly investigate the patients with recurrent lower respiratory infections or compressive symptoms to rule out acute exacerbations, cancer or mass lesions and possible underlying tracheobronchomegaly, respectively [1-7]. It is mostly idiopathic with most of the cases identified as congenital but it can be acquired too. Some cases were also described in association with the connective tissue diseases [1,5]. There was no obvious correlation found between smoking and the development of this condition. Similarly, our patient was a non-smoker and yet had this syndrome with the newly diagnosed SCC of the lung that was impinging on the superior vena cava and trachea.

Mounier-Kuhn syndrome can be classified into mild, moderate or severe categories based on the symptomatology [1]:

1- Mild: Patients are usually asymptomatic and do not require further treatment. Nevertheless, they should have yearly follow-ups and receive their routine flu shots and pneumococcal vaccines.

2- Moderate: These patients have recurrent upper and lower respiratory tract infections. They usually manifest with hemoptysis, spontaneous pneumothorax or respiratory failure and require supplemental oxygen, aggressive physical therapy and postural drainage techniques. Antibiotics should be promptly started to prevent infections. In certain cases, bronchoscopy may also be needed.

3- Severe: This group of patients carries a very poor prognosis and is usually identified on autopsy. These patients generally present at a very late stage with an impending respiratory failure or severe hypoxia and an early mortality.

An evidently increased tracheal diameter on a chest radiograph should raise the suspicion of Mounier-Kuhn syndrome but it is not a very sensitive and specific modality for diagnosing the condition. The gold standard for its diagnosis is the biphasic CT imaging that most effectively measures the transverse and sagittal diameters of the trachea and bronchi. The generally accepted diagnostic criteria for this syndrome is the transverse diameters of the trachea, right and left bronchi exceeding 30 mm, 20 mm and 18 mm, respectively [6,9]. Patients suspected to have this disorder or signs and symptoms suggestive of obstructive pulmonary lung disease, e.g., wheeze, chronic productive cough, etc. should also undergo PFTs. Fiberoptic bronchoscopy can also be used to detect the aforementioned pathological processes. It can also identify an occult mass or cancer contributing to the exacerbation of symptoms by taking biopsy samples. Treatment options for this syndrome are limited. Tracheal stenting has shown promising results in patients with multiple tracheal diverticuli and advanced stages. There is usually no role for surgery because of the diffuse nature of the disease [9]. However, bilateral lung transplantation was successfully executed and can be considered as a treatment option for the severe cases [10].

## Conclusions

Mounier-Kuhn syndrome, despite being rare, can prove to be quite fatal, if not investigated and managed properly. In our patient, the major concern was the timely evaluation and decision for stenting. Since he was already having dyspnea secondary to metastasis from SCC that led to tracheal narrowing and any delay or ineffective monitoring could have led to respiratory failure or coma secondary to cerebral edema. Hence, this patient, already a known case of Mounier-Kuhn syndrome, gave us an entirely different dimension to ponder upon, that though it is mostly seen in conjunction with recurrent infections but an associated primary or metastatic cancer can also add to the problems if not addressed on the right time and with the appropriate approach. Malignancy should, therefore, be included as a potential complication of this syndrome.
